# Irreversible electroporation on the small intestine

**DOI:** 10.1038/bjc.2011.582

**Published:** 2012-01-05

**Authors:** M A Phillips, R Narayan, T Padath, B Rubinsky

**Affiliations:** 1 Department of Mechanical Engineering, University of California-Berkeley, 6124 Etcheverry Hall, Berkeley, CA 94720, USA; 2 Pathology Research Laboratory, Inc., 2829 Depot Road, Suite 4, Hayward, CA 94545, USA

**Keywords:** irreversible electroporation, abdominal cancer, tissue recovery

## Abstract

**Background:**

Non-thermal irreversible electroporation (NTIRE) has recently been conceived as a new minimally invasive ablation method, using microsecond electric fields to produce nanoscale defects in the cell membrane bilayer and induce cell death while keeping all other molecules, including the extracellular matrix, intact. Here, we present the first *in vivo* study that examines the effects of NTIRE on the small intestine, an organ whose collateral damage is of particular concern in the anticipated use of NTIRE for treatment of abdominal cancers.

**Methods:**

A typical NTIRE electrical protocol was applied directly to the rat small intestine and histological analysis was used to examine the effect of NTIRE over time.

**Results:**

The application of NTIRE led to complete cell ablation in the targeted tissue, but the animal did not show any physiological effects of the procedure and the intestine showed signs of recovery, developing an epithelial layer 3 days post treatment and regenerating its distinct layers within a week.

**Conclusion:**

Our results indicate that this novel procedure can be used for abdominal cancer treatment while minimising collateral damage to adjacent tissues because of the unique ability of the NTIRE ablation method to target the cell membrane.

Non-thermal irreversible electroporation (NTIRE) is a new minimally invasive surgical technique that was originally conceived from theoretical considerations with the capability of selectively targeting cell membranes to treat biological tissues (Davalos *et al*, 2005). Rather than using drug-induced chemical selectivity, NTIRE is based on fundamental biophysical principles. The cell ablation technique used in this study deals with a bioelectric and a biothermal phenomenon. The bioelectric phenomenon is characterised by the permeabilisation of the cell membrane's lipid bilayer through the application of very brief (nanosecond to millisecond), high field (in the range of MV m^−1^) electric pulses across the cell (Weaver and Chizmadzhev, 1996; Weaver, 2000; Chen *et al*, 2006). This biophysical phenomenon has been observed for centuries (Nollet, 1754) and studied intensively since the mid 1900s (e.g., see Sale and Hamilton, 1967). Several different names have been used in literature to describe this phenomenon; electropermeabilisation is used to describe the physical effect of the pulses on the cell membrane (Stopper *et al*, 1985), and electroporation describes the hypothetical pores that form (Neumann *et al*, 1982). The effects of electroporation depend on the magnitude and duration of the pulsed electric field as well as other factors, such as cell size and shape and number of electrical pulses applied. The electric field magnitude triggers pore formation (Teissie and Rols, 1993), whereas the pulse length influences the pore expansion process (Gabriel and Teissie, 1997). The family of electrical pulses that cause electroporation are divided into two types; in reversible electroporation, the cells survive the permeabilisation process, and irreversible electroporation results in cell death because of the lipid bilayer destabilisation and permeabilisation (Weaver and Chizmadzhev, 1996; Weaver, 2000; Chen *et al*, 2006). Physical principles indicate that the energy dissipation of high electric fields such as those involved in electroporation can lead to an increase in tissue temperature due to Joule heating (Chang and Nguyen, 2004). Indeed these thermal effects have been used clinically with such applications as radiofrequency, microwave, laser, high frequency ultrasound, and even conventional electric heating ablation (Davalos *et al*, 2005). Such elevated temperatures, however, ablate tissue by denaturation of all the molecules in the treated volume. This biothermal effect depends on the electrical parameters; it can elevate the tissue temperature to levels at which the cells become damaged, or it can result in only slight temperature increases that do not cause thermal damage to occur (Lavee *et al*, 2007). We have found that within the family of electric fields that cause irreversible electroporation, there is a subset that minimises Joule heating, resulting in temperature increases that stay below the threshold for thermal damage (Davalos *et al*, 2005). To be succint, we will refer to this subset of electric fields as ‘Non-Thermal Irreversible Electroporation’ or NTIRE, designating electric fields that cause irreversible electroporation to occur without resulting in a level of elevated temperatures that can induce thermal damage.

Though this biophysical phenomenon is not yet completely understood (Teissie *et al*, 2005) electroporation is becoming extensively used in biotechnology and medicine. In the reversible mode, electroporation has become a central technology for cell manipulation (Richter *et al*, 1981; Neumann *et al*, 1982), and, in combination with chemicals, it is considered promising for gene therapy (Titomirov *et al*, 1991; Heller and Heller, 2010), and is also used clinically for electrochemotherapy (Snoj *et al*, 2007; Gargiulo *et al*, 2010). We hypothesised that if certain electric pulses could be found that can irreversibly permeabilise the cell membrane without elevating the affected tissue temperature to levels that may induce thermal damage, then large volumes of tissue could be treated using NTIRE (Davalos *et al*, 2005). Our first mathematical study has proven that, while limited in range, such a domain of electric fields exists (Davalos *et al*, 2005). Our subsequent studies have shown that NTIRE can ablate tissue (Edd *et al*, 2006; Rubinsky, 2007; Rubinsky *et al*, 2007) while retaining the structural integrity of blood vessels, nerves, and extracellular matrix (Onik *et al*, 2007; Phillips *et al*, 2010) and that it is effective in destroying cancer in animal models (Al-Sakere *et al*, 2007; Ellis *et al*, 2011). Non-thermal irreversible electroporation involves the insertion of thin needle electrodes around an undesirable tissue or cell mass and the application of brief microsecond-scale electric pulses. The ability to apply NTIRE in a minimally invasive manner and the safety of this procedure (Thomson *et al*, 2011) has led to a recent surge in its clinical use. Although long-term studies show evidence that NTIRE spares tissue scaffolds and blood vessel structure, thus far there has been no systematic study on how NTIRE affects critical tissues such as the small intestine or the process of tissue regeneration.

One emerging application of NTIRE is in relation to treatment of abdominal cancer and the ability to avoid collateral damage even in tissues within the electric field. In this work, we chose to study the effect of the NTIRE on a body organ that is very often subject to collateral damage in minimally invasive or non-invasive surgery: the small intestine. For instance, collateral damage to the small intestine often occurs after radiotherapy for pelvic or abdominal malignancies as well as a side effect of chemotherapy, resulting in bloating, abdominal cramping, severe diarrhea, nausea, and vomiting (Keefe *et al*, 2000; Ciorba and Stenson, 2009; Han *et al*, 2011). These side effects are seen as the limiting factor in increasing both chemotherapy and radiotherapy dosage and can force discontinuation of treatment (Keefe *et al*, 2000; Packey and Ciorba, 2010). The small intestine may be especially susceptible to these treatment methods as it experiences a high cell turnover rate, especially for the rapidly dividing cells of the mucosa (Keefe *et al*, 2000). Our hypothesis is that, due to the ability of NTIRE to spare the extracellular matrix, the intestine will remain structurally intact after treatment with NTIRE, survive the treatment, and recover. This study was performed in a small animal model in which we studied the effects of applying a typical NTIRE protocol directly to the intestine.

## Materials and Methods

### Finite element modelling of electrical parameters to predict thermal damage

In order to choose electrical parameters for experimental use that would not cause extensive heating and thermal damage to the tissue, a transient finite element analysis was performed, modelling the effect of Joule heating on the temperature distribution in the intestinal tissue. The results were then used to determine the accumulated thermal damage in the tissue over time and to ensure that the electrical parameters modelled would minimise thermal damage to the tissue. A commercial finite element package (Comsol Multiphysics 3.5a) was used to develop the model and plan the electrical treatment parameters. The small intestine and plate electrodes were modelled two-dimensionally as a 4.63 × 1-mm^2^ rectangle pressed between two stainless steel electrodes (each of 9.4 × 15.6 mm^2^) and held within a 5 × 5-cm^2^ airspace. The small intestine's dimensions were based on experimental observations as well as data from literature (Dou *et al*, 2002). The plate electrodes and small intestine are held close to the body during the procedure, and thus, the system was modelled as being surrounded by air at an elevated temperature of 37°C. The thermal and electrical properties of the small intestine were assumed to be both isotropic and homogeneous in cross-section. This model followed the analysis described by Phillips *et al* (2011). Briefly, the Laplace equation (*σ*∇^2^*ϕ*=0) was solved in order to determine the heat generation per unit volume due to Joule heating (*q*_*JH*_):



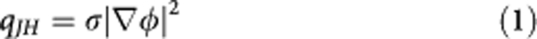



where *ϕ* is the electric potential and *σ* is the electrical conductivity. The top electrode was set as having a positive potential (*ϕ*_1_=*V*_o_) and the bottom electrode was set as ground (*ϕ*_2_=0), where *V*_o_ is the potential difference applied across the electrodes. The boundaries between the electrodes and air and between the small intestine and air were set as electrically insulating. The resulting heat generation term (*q*_*JH*_) was then used as the heat source term in the heat conduction equation in order to solve for the temperature distribution in the tissue.



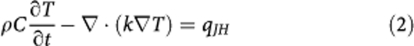



Here *ρ* is the material density, *C* is the heat capacity, and *k* is the thermal conductivity. The entire system was initially held at the physiological temperature of 37°C, and the edges of the air space were held at 37°C, providing a conservative overestimate of the temperature.

In this model, the full procedure used 50 square dc pulses of 70 *μ*s each and a pulse frequency of 4 Hz. Electrical and thermal properties used for the tissue are as follows: *σ*=0.6 S m^−1^ (Gabriel *et al*, 1996), *C*=3750 J (kgK)^−1^ (Davalos *et al*, 2003), *ρ*=1000 kg m^−3^ (Davalos *et al*, 2003), and *k*=0.5 W mK^−1^ (Davalos *et al*, 2003), and the electrodes were modelled using the properties of stainless steel. The temperature increases during each pulse due to the resistive heating and is dissipated due to conduction to the electrodes and to the surrounding air. In order to solve for the temperature distribution over the entire procedure and thus find a measure of the resulting thermal damage to the tissue, Matlab R2009b (MathWorks, Natick, MA, USA) was used to run Comsol Multiphysics 3.5a. The coupled electric field and heat conduction equations were solved at the end of each pulse and after each interval between pulses. The maximum tissue temperature at each time step was stored as well as once every second for 3 min after the last pulse. The maximum temperature values were then used to calculate the thermal damage to the tissue using the Henriques and Moritz thermal damage integral (Diller and Pearce, 1999):



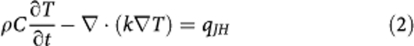



where *t* is the time in seconds, *R* is the ideal gas constant, *A* is the measurement of molecular collision frequency, and Δ*E* is the activation energy for the molecules to denature. *A* and Δ*E* are typically determined experimentally. As no values could be found in the literature specifically for small intestinal tissue, values determined for arterial tissue (Agah *et al*, 1994; Wright, 2003) were used here in order to gain a rough estimate of the potential thermal damage, giving *A*=1.552 × 10^67^ s^−1^ and Δ*E*=4.3 × 10^5^ J mol^−1^. Ω is the damage parameter and can be expressed as the logarithm of the ratio of the undamaged molecules before the procedure to the undamaged molecules at a given time. Thus, calculating Ω can give an estimate of the percentage of thermal damage that occurs throughout the procedure. Equation (3) was applied to the entire electroporation procedure, giving a thermal damage parameter of Ω=0.0015, corresponding to ∼0.15% damage at the location of maximum temperature increase. The maximum tissue temperature obtained throughout the entire procedure was 39.45°C. This model shows very little temperature increase to the small intestine tissue during the electroporation procedure because of the large size of the stainless steel electrodes in comparison to the small intestine tissue. Thus, most of the heat was quickly conducted to the electrodes. As this model predicts very little damage while incorporating assumptions that would actually over predict tissue temperature (over predictions include two-dimensional model, ignoring heat loss due to natural convection, and using the maximum tissue temperature to obtain the damage parameter), it was determined that the parameters modelled could be used experimentally without causing thermal damage to the majority of the tissue *in vivo*.

### *In vivo* experimental procedure

Thirteen Sprague–Dawley rats weighing 200–300 g were used in this study. All animals received humane care from properly trained professionals in compliance with both the Principals of Laboratory Animal Care and the Guide for the Care and Use of Laboratory Animals, published by the National Institute of Health (NIH publication no. 85-23, revised 1985).

Animals were anaesthetised with 2 mg kg^−1^ meloxicam followed by chamber induction with isoflurane. Anaesthesia was administered throughout the procedure with vaporised isoflurane. The depth of anaesthesia was assessed before surgery and throughout the surgical procedure. After the level of anaesthesia was verified, the abdominal skin was shaved and an antiseptic was applied. Sterile surgical techniques were used throughout the entire surgery. Lidocaine (up to 7 mg kg^−1^) was administered subcutaneously along the midline of the abdomen as a local anaesthesia. A 3-cm midline abdominal incision was made, exposing the small intestine. A set of plate electrodes (BTX Caliper Electrode, Harvard Apparatus, Holliston, MA, USA) was gently applied across the ileum, about 5 cm proximal to the ileo-cecal valve. The measured distance between the two electrodes was approximately 1 mm and was consistent for all animals tested. A sequence of 50 DC pulses of 200 V (corresponding to an electric field of ∼2000 V cm^−1^), 70 *μ*s each, and a frequency of 4 Hz was applied between the electrodes using a high voltage pulse generator designed for electroporation procedures (ECM 80, Harvard Apparatus). The electrical parameters used in this study are typical to those used in clinical procedures to produce irreversible electroporation without causing thermal damage to the intestinal tissue. The procedure was repeated, using two successive locations along the ileum and treating approximately 1.9 cm along the length. The location of treatment was noted based on anatomy, and a suture knot was placed in the mesentery to mark the IRE-treatment zone. At the end of the experiment, the abdomen wall was sutured closed, followed by the skin incision. Tissue adhesive was applied over the skin sutures. Buprenorphine (0.05 mg kg^−1^) was administered as an analgesic following the procedure. Animals were divided into three groups of four animals each and were kept alive for 1, 3, or 7 days before being euthanised.

During the first 24 h after surgery, the animals were given two additional doses of buprenorphine (0.05 mg kg^−1^) and meloxicam (2 mg kg^−1^), spaced out over 8 h increments. After surgery, animals were checked daily to ensure that they recovered, stayed healthy, and were not experiencing pain. Symptoms that were monitored included reduced food intake, fever, hunched posture, lack of grooming or locomotion, swelling around the incision, facial discharges around the nose and eye, and diarrhea. All animals were also weighed daily.

Animals were euthanised by a combination of an overdose of vaporised isoflurane and a bilateral chest dissection while under a deep anaesthesia induced by an intraperitoneal injection of ketamine (90 mg kg^−1^) and xylazine (10 mg kg^−1^). The treated regions of the small intestine as well as untreated sections 3–5 cm proximal and 3–5 cm distal of the treated region were harvested. Each intestinal segment was flushed with saline, fixed with 10% buffered formalin, and submitted to an independent pathology lab (Pathology Associates, Inc., Berkeley, CA, USA). The samples were embedded in paraffin and sectioned with a microtome (5-*μ*m-thick). All samples were cut perpendicular to the intestinal axis, exposing the ileum's cross-section. Each sample was stained with haematoxylin and eosin. Selected samples from each group were cut in cross-section and stained with Masson's trichrome to examine the structure of the extracellular matrix.

Examination of each section was focused on the small intestine's cellular and extracellular response to NTIRE over time.

## Results

Thirteen Sprague–Dawley rats were used in this study. One animal was lost during surgery due to an overdose of isoflurane. All other animals recovered quickly from the surgical procedure and remained active, maintaining weight over the 1- to 7-day period. Normal eating habits and stool were observed. Five of the animals experienced slight porphyrin staining around the eyes after surgery that cleared up on its own within 24 h. Otherwise, the animals did not display any of the typical signs of pain, and observations indicated that the animals did not experience any adverse effects due to the NTIRE treatment procedure.

**Figure 1:**
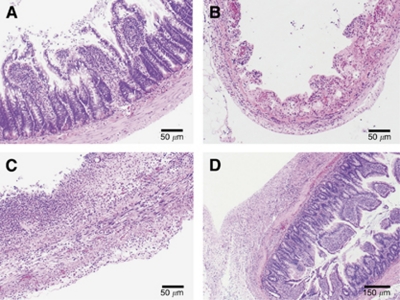
The effect of NTIRE on the small intestine. (**A**) The untreated control shows a typical, healthy small intestine. (**B**) One day after NTIRE treatment, the small intestine shows complete cellular ablation. (**C**) Treated areas 3 days after treatment still depict a loss in the structural layers of the cell. (**D**) At 7 days after applying the NTIRE protocol to the small intestine, the distinct structure of the small intestine is seen.

Histological analysis of the small intestine 1, 3, and 7 days was used to examine the effect of NTIRE on the small intestine over time. At day 1, ileum segments exhibited severe necrotic tissue with complete obliteration of cellular architectural details. At 3 days after treatment, the structure of the small intestine was still necrotic. At 7 days, however, the ileum appeared to have regained much of its structure and showed distinct tissue layers such as the mucosa, submucosa, muscular layers, and serosa. This can be seen in Figure 1.

The results 1 day after NTIRE treatment (Figure 1) show that the irreversible electroporation protocol was strong enough to affect all layers of the small intestine. Here, a complete loss of intestinal epithelium cellular architectural detail can be seen, and the villi are losing organisation and form. Though acute necrotic tissue was observed along the entire circumference of the NTIRE-treated regions, no perforations were observed, indicating that the structure of the small intestine was still intact enough to keep fissures from forming and the luminal contents from spilling outward.

**Figure 2:**
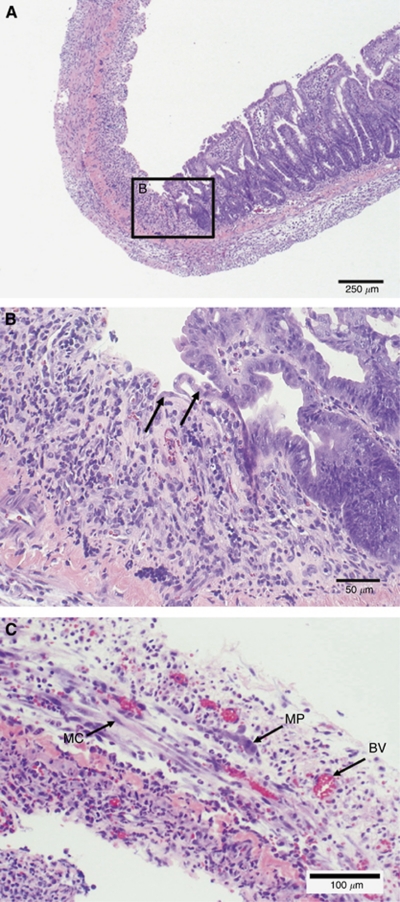
Small intestine 3 days post NTIRE. (**A**) The interface between an NTIRE-treated region and an untreated region of the small intestine is shown. (**B**) A closer look with higher magnification reveals immature epithelial cells that can be seen migrating into the NTIRE-treated zone, as highlighted by the arrows. (**C**) The presence of blood vessels (BV), the myenteric plexus (MP), and myocytes (MC) can also be seen.

The original villi are completely obliterated at 3 days after applying NTIRE (Figures 1 and 2). Here, though the extracellular structure still exists, the tissue is void of the proper cellular structure and tissue layers. Signs of tissue repair, however, are evident. A new epithelial layer can be seen forming along the edges of the treated zones, as indicated by the appearance of immature epithelial cells. In addition, blood vessels and nerve bundles are present and regenerating myocytes can also be seen.

**Figure 3:**
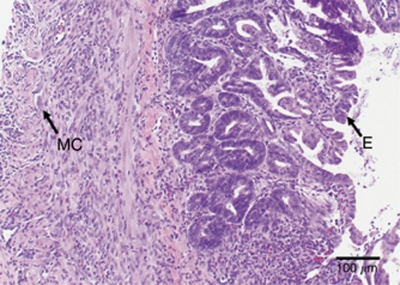
Small intestine 7 days post NTIRE. The small intestine is beginning to regain its cellular structure 7 days after NTIRE treatment and the mucosa has regenerated, as indicated by the presence of new villi lined with epithelial cells (E). The muscularis is also becoming repaired with immature muscle cells (MC).

At 7 days post NTIRE, the tissue structure appears to have recovered into its distinct layers (Figure 3). The mucosa is in the process of organisation, and normal repair and replacement are occurring. The immature, frond-shaped villi are lined with epithelial cells, and immature muscle cells are now present in the muscle layers. Regenerating granular cells are also present.

**Figure 4:**
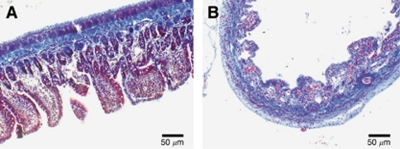
Effect of NTIRE on cell scaffold structure. Although 1 day after NTIRE treatment, there is a loss of cellular architecture throughout the intestine (**B**) as compared with the control (**A**), the cell scaffold remains intact. The blue collagen fibers are similar in morphology after NTIRE treatment when compared with the control.

Masson's trichrome stain was also used on select intestinal samples in order to examine the effect of NTIRE on the extracellular matrix. Here, an NTIRE-treated sample harvested 1 day after the procedure is compared with the control (Figure 4). The collagen fibers are stained blue, muscle fibers are stained red, and cell cytoplasm and nucleus are stained light red and dark brown, respectively. Though the cellular makeup of the intestinal tissue is strongly affected by the NTIRE treatment procedure, it can be observed that the extracellular makeup is very similar in morphology between the treated and untreated samples, indicating the extracellular architecture is still intact.

## Discussion

In this study, a typical NTIRE electrical pulse protocol was applied across the small intestine, in order to assess the tissue's ability to respond and recover to the NTIRE treatment. Though the plate electrodes used in this study are not the method of applying NTIRE clinically, they are convenient for inducing a pulsed electric field across the tissue in a lab setting, enabling one to study the direct effect of the electrical protocol on the tissue's ability to recover. At one day after treatment, the small intestine saw complete cellular ablation throughout its entire circumference, indicating that the electrical parameters chosen were strong enough to cause irreversible electroporation throughout all layers of the tissue. For this study, we wished to provide complete damage to the tissue by electroporation while avoiding any effects of thermal damage. Using finite element modelling, the electrical parameters were chosen such that they would be well above the threshold for irreversible electroporation without resulting in thermal damage due to Joule heating to the tissue. As is evident in Figure 1, the cellular destruction to the tissues is complete and a full loss of cellular architectural detail can be seen. However, as the extracellular matrix is not affected by NTIRE, the structural integrity of the small intestine remained.

Despite complete obliteration of the cellular structure, the tissue showed signs of recovery. The modality of cell death due to NTIRE occurs quickly (Lavee *et al*, 2007), and though the small intestine villi and crypt were completely destroyed, signs of tissue repair are already evident 3 days post treatment (Figure 2). The crypts contain multipotent stem cells, which differentiate and move up the villi, replacing cells that slough off in normal, healthy tissue every 1–3 days (Dignass, 2001; Ciorba and Stenson, 2009). Though the cells within the crypts are ablated within the treated area, it appears that immature epithelial cells are being produced from the edges of the treated zones and are able to migrate inward, producing a new epithelial cell layer. In addition, it can be seen that the framework of the muscularis is preserved at both 3 and 7 days after NTIRE (Figures 2 and 3). Tissue recovery continues at 7 days post-IRE, where repair is evident and the tissue appears to have regained its distinct layers (Figure 3). Normal repair and replacement of the mucosa, submucosa, and muscularis is occurring. Though additional studies are needed in order to assess tissue function and investigate the effects of NTIRE on the intestine over a longer time course than 7 days, it is evident here that the small intestine was able to go from complete cellular destruction to regeneration of intestinal layers and villi within 1 week. Longer-term studies are planned in order to assess the continued recovery of the small intestine.

NTIRE specifically targets the cell membrane, allowing for the preservation of tissue structural components such as the extracellular matrix, blood vessels, and nerves (Onik and Rubinsky, 2010; Phillips *et al*, 2010). It can be seen that this holds true for the small intestine as well. Masson's trichrome staining of the ileum at 1 day after NTIRE treatment illustrates that the extracellular matrix is still intact (Figure 4). Lymphatic supplies, nerves, and blood cells are still functioning, providing a framework for epithelisation that can be observed at 3-days post NTIRE treatment. This framework allows for restoration of the blood supply, as seen for the 3- and 7-day treatment groups. Thermal coagulation and thrombosis to the blood vessels has not occurred, and the capillaries are open and blood is flowing (Figure 2), resulting in presence of immature villi and granular cells 7 days after electroporation. It is hypothesised that the ability of NTIRE to preserve important structures such as the extracellular matrix, blood vessels, and nerves greatly aids in the overall recovery of the small intestine.

As illustrated both here and in the literature (Rubinsky, 2007; Onik and Rubinsky, 2010), NTIRE preserves the tissue vasculature, as compared with the vascular damage that can result from ionising radiation (Packey and Ciorba, 2010). Vascular damage occurs during ionising radiation (Demirer *et al*, 2007), and some believe that this damage can lead to complications with the small intestine years after treatment (Paris *et al*, 2001; Packey and Ciorba, 2010). Though NTIRE does cause endothelial cell death, vessel occlusion does not occur (Onik and Rubinsky, 2010), and endothelial cells have been shown to reline the blood vessels within a week of NTIRE treatment (Phillips *et al*, 2011), leaving an intact and functioning micro- and macrovasculature. Though long-term studies would be needed in order to determine what effects NTIRE has on the small intestine years after treatment, it is believed here that the unique ability of NTIRE to preserve blood vessels and the extracellular matrix not only aids in short-term recovery, but could also protect the tissue from developing the long-term complications often seen from radiation treatments.

NTIRE is viewed as a promising modality for cancer treatment. Because of its ability to preserve important structural and functional aspects of the tissue while specifically targeting the cell membrane, NTIRE may be a promising alternative for treating malignant tumors located near sensitive organs. For example, ablating abdominal tumors could cause damage to small intestine. The goal of this study was to evaluate the ability of the small intestines to survive direct application of NTIRE. For this study, 2000 V cm^−1^ were applied directly to the small intestine, resulting in complete cellular ablation 1 day after treatment. The extracellular matrix, blood vessels, and nerves, however, were preserved, aiding in recovery of the tissue. By 3 days after treatment, the epithelial layer had begun to recover, and the 7-day group showed regeneration of the villi and a restored structural layers including the mucosa, submucosa, and muscularis. Although substantial further investigation is needed, this pilot study indicates that the high turnover rate of the small intestine mucosa coupled with the ability of NTIRE to preserve the extracellular matrix and other important functional structures allows for a quick recovery of the intestine after electroporation treatment. This study predicts that, should the small intestine be within the electric field generated while treating an abdominal tumor with NTIRE, the intestine will be able to heal and regenerate.
